# Artificial intelligence in ARDS: From automated support to personalized ventilation

**DOI:** 10.1016/j.jointm.2025.10.003

**Published:** 2025-12-02

**Authors:** Javier Muñoz, Nerio José Fernández-Araujo, Rocío Ruíz-Cacho, Javier Muñoz-Visedo

**Affiliations:** 1 Department of Intensive Care Medicine, Hospital General Universitario Gregorio Marañón, Madrid, Spain; 2 Instituto de Investigación Sanitaria Gregorio Marañón (IiSGM), Madrid, Spain; 3 Escuela Superior de Ciencias Experimentales y Tecnología, Universidad Rey Juan Carlos, Madrid, Spain

## Introduction

Mechanical ventilation, a cornerstone of critical care, still relies on standardized protocols that frequently overlook patient variability and the nonlinear nature of respiratory physiology. Artificial intelligence (AI) is emerging as a tool to support decisions, detect patient–ventilator interactions, and personalize support.^[^^1^^]^ Nonlinearities such as pressure–volume hysteresis, time-varying compliance, and dynamic airway resistance complicate the prediction of optimal responses; AI systems address these complexities by recalibrating predictions against breath-by-breath data, enabling physiologically grounded adaptation.^[^^2^^]^ Although AI-driven ventilation strategies apply to various forms of respiratory failure, most validation efforts and clinical advances have occurred in patients with acute respiratory distress syndrome (ARDS), the prototypical model of complex and heterogeneous lung mechanics. By integrating multimodal signals, AI has the potential to move beyond static guidelines, bridging the gap between heterogeneous physiology and real-time decision-making.^[^^1^^,^^2^^]^

## The Current Role of AI in Mechanical Ventilation

Current applications of AI focus primarily on asynchrony detection, clinical decision support, and prediction of weaning readiness.^[^[3], [4], [5], [6]^]^ Convolutional neural networks (CNNs) and recurrent neural network (RNNs) are typically trained on high-resolution ventilator waveforms – pressure, flow, and volume – sometimes complemented by diaphragmatic activity (EAdi) or oxygen saturation (SpO₂) to improve detection accuracy.^[^^7^^,^^8^^]^ Weaning readiness is commonly defined as successful extubation without reintubation within 48 h, using criteria such as respiratory rate, tidal volume, and oxygenation indices.^[^^6^^,^^9^^]^ Predictive models have outperformed traditional scores, providing earlier and more reliable identification of patients ready for liberation from ventilation.^[^^6^^]^ Beyond waveform analysis, AI-based decision support tools integrate demographic, physiological, and laboratory data to recommend ventilator settings. These systems may reduce workload, standardize practice, and anticipate complications, though most remain proof-of-concept.^[^^5^^,^^10^^]^ Illustrative examples of AI applications in mechanical ventilation are summarized in Table 1.

**Table 1:** Illustrative applications of artificial intelligence in mechanical ventilation.

Author	Approach/model	Clinical focus	Main input data	Reported performance/outcome	Design/setting
Lee et al.^[7]^; Park et al.^[9]^	CNN/deep learning	Asynchrony detection and weaning prediction	Ventilator waveforms; diaphragmatic EMG (EAdi)	CNN improved signal classification for synchrony; AUROC=0.91 for weaning readiness	Retrospective datasets (MIMIC-IV, ventilator signals)
Liu et al.^[5]^	Reinforcement learning	Optimization of ventilator settings (PEEP, VT, FiO₂)	Waveforms + EHR time-series	Simulated policies suggested increased ventilator-free days	Retrospective; multicenter public databases (MIMIC-IV, eICU-CRD)
Stivi et al.^[6]^	Systematic evidence	Prediction of weaning success	Clinical and ventilatory parameters	Pooled sensitivity 0.82; specificity 0.78 across models	Systematic review of 18 studies
Chen et al.^[10]^; Zaidi et al.^[13]^; Cappellini et al.^[14]^	Physiological integration	PEEP titration; driving pressure monitoring; EIT-guided adjustment	Recruitment-to-inflation ratio, ΔP, EIT aeration maps	R/I ratio associated with recruitability; ΔP linked to mortality; EIT used to infer lung aeration and circulation	Prospective trials (Chen et al.^[10]^), narrative and integrative reviews

Examples highlight different methodological approaches (deep learning, reinforcement learning, systematic evidence, and physiological integration) and their clinical focus, input data, performance, and study design.

ΔP: Driving pressure; AUROC: Area under the receiver operating characteristic curve; CNN: Convolutional neural network; EAdi: Electrical activity of the diaphragm; EHR: Electronic health records; eICU-CRD: eICU Collaborative Research Database; EIT: Electrical impedance tomography; EMG: Electromyography; FiO_2_: Fraction of inspired oxygen; MIMIC-IV: Medical Information Mart for Intensive Care IV; PEEP: Positive end-expiratory pressure; R/I ratio: Recruitment-to-inflation ratio; VT: Tidal volume.

## Strengths and Limitations of AI for Mechanical Ventilation

AI can process vast and heterogeneous data, uncover hidden patterns, and reduce clinician workload. Yet, the dynamic physiology of mechanical ventilation requires real-time adaptation. Practical implementation demands subsecond resolution so that algorithms can respond within a single breath cycle.^[^^2^^]^

Beyond speed, robustness and transparency are critical. Fuzzy logic systems, unlike binary rule-based approaches, allow graded outputs (e.g., “moderate effort”) that improve flexibility. Compared with machine learning (ML) models, fuzzy systems are easier to interpret but require expert-defined membership functions; clinical studies have reported that fuzzy controllers can shorten weaning time compared with standard approaches, while ML-based models achieve higher predictive accuracy at the cost of reduced interpretability.^[^^1^^,^^3^^,^^5^^,^^6^^]^

Data limitations remain a barrier. Many models are trained on single-center datasets, risking overfitting and poor generalizability. Public databases such as Medical Information Mart for Intensive Care IV (MIMIC-IV) and eICU Collaborative Research Database (eICU-CRD) illustrate these issues: waveforms may be available in one but not the other, gas exchange variables use heterogeneous units, and timestamps differ in granularity. Such inconsistencies complicate validation.^[^^5^^,^^8^^,^^11^^]^

Finally, translation requires ethical safeguards and resilience against failure. Fault-tolerant designs can include redundancy – such as combining waveform and vital sign analysis – and fallback protocols that revert to rule-based recommendations if noise or data loss occurs. Explainable AI approaches are also increasingly important to enhance the interpretability of model outputs.^[^^11^^,^^12^^]^

## Bridging Physiology and Machine Learning

AI should embed physiological principles rather than rely solely on statistical correlations. Hybrid models that incorporate explicit rules can improve interpretability and constrain outputs to physiologically plausible ranges.^[^^1^^,^^13^^]^ For example, bounding tidal volume within 6–8 mL/kg of predicted body weight or maintaining plateau pressure <30 cmH_2_O provides guardrails aligned with protective strategies.^[^^13^^]^ Similarly, recruitment-to-inflation ratios can limit positive end-expiratory pressure (PEEP) optimization to reasonable levels.^[^^10^^]^ Electrical impedance tomography (EIT) generates real-time aeration maps that guide PEEP titration.^[^^14^^]^ When combined with deep learning, EIT has been used to infer respiratory and circulatory parameters, and convolutional models applied to diaphragmatic electromyogram signals have improved neurally adjusted ventilatory assist systems by enhancing synchrony.^[^^7^^]^

These examples show how AI can couple with monitoring to translate physiology into ventilatory adjustments. Mechanistic integration also involves using dynamic variables such as compliance trends, oxygenation indices, or asynchrony index to guide real-time decisions, while static features such as ARDS phenotype or comorbidities provide baselines.^[^^6^^,^^9^^,^^11^^]^ Finally, subphenotypes of ARDS provide a mechanistic layer for personalization: hyperinflammatory patients, characterized by higher recruitability, may benefit from higher PEEP, while hypoinflammatory patients may be harmed by aggressive strategies. AI classifiers trained on these profiles illustrate how linking biological signals with mechanics can anchor personalization in pathophysiological principles.^[^^15^^]^

## From Automation to Personalization

**Figure 1:**
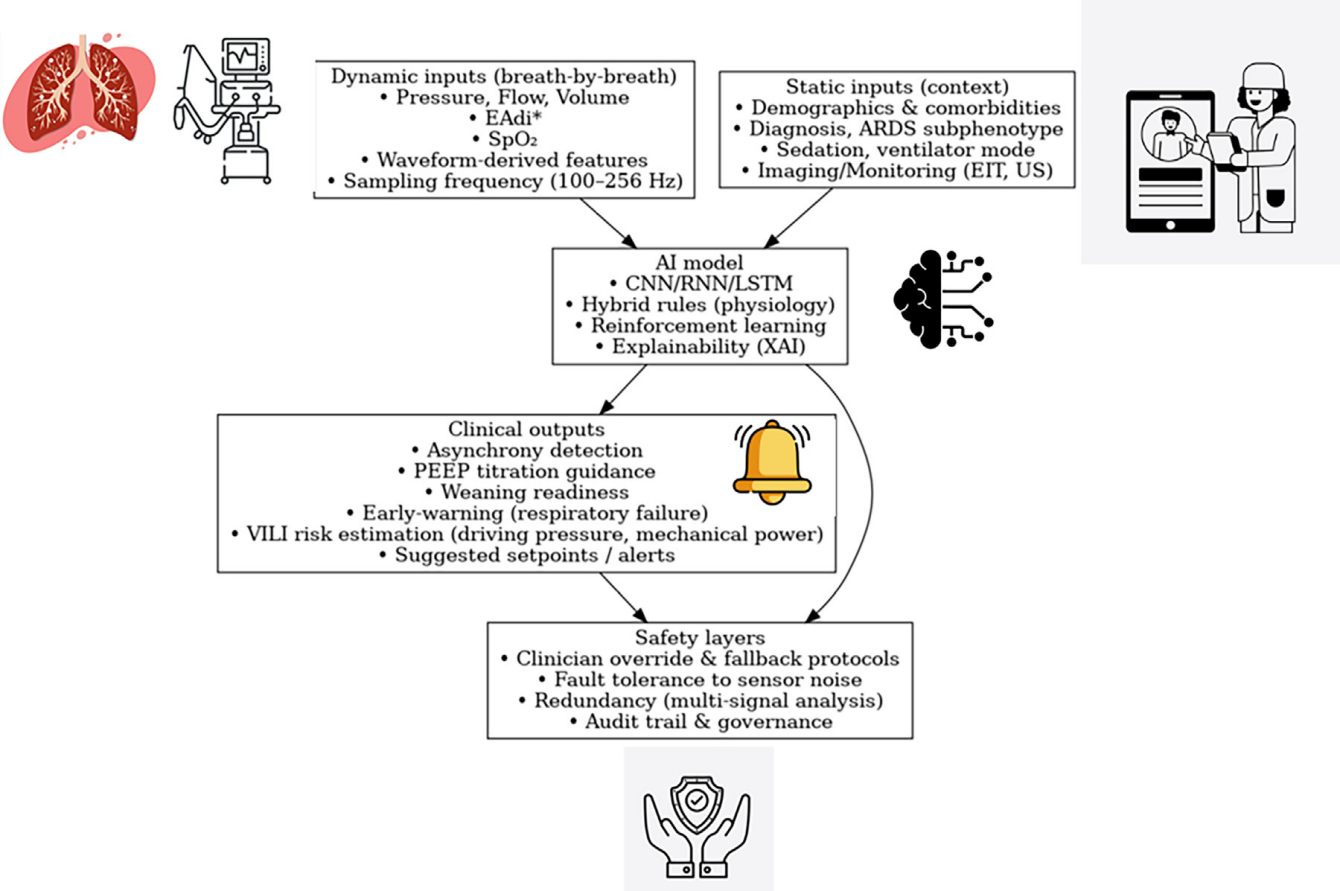
Conceptual framework for artificial intelligence in mechanical ventilation. This diagram illustrates the integration of multimodal inputs (dynamic breath-by-breath data and static contextual features) into an AI model incorporating CNN/RNN/LSTM, hybrid physiological rules, reinforcement learning, and XAI. Outputs include clinical recommendations such as asynchrony detection, PEEP titration, weaning readiness, early warning of respiratory failure, VILI risk estimation, and suggested setpoints/alerts. Safety layers ensure clinician override, fault tolerance via multisensor analysis, and audit trails for governance. * EAdi refers to the electrical activity of the diaphragm, a signal obtained through neurally adjusted ventilatory assist (NAVA) monitoring. AI: Artificial intelligence; ARDS: Acute respiratory distress syndrome; CNN/RNN/LSTM: Convolutional neural networks/recurrent neural networks/long short-term memory; EAdi: Electrical activity of the diaphragm; EIT: Electrical impedance tomography; PEEP: Positive end-expiratory pressure; SpO_2__:_ Oxygen saturation; US: Ultrasound; VILI: Ventilator-induced lung injury; XAI: Explainable artificial intelligence.

AI is shifting from automation to true personalization of ventilation.^[^^6^^,^^15^^]^ The overall interaction between AI systems, physiological variables, and ARDS phenotypes is summarized in Figure 1. Unsupervised clustering of large intensive care unit (ICU) datasets has identified ARDS subphenotypes with heterogeneous responses: hyperinflammatory patients, with greater recruitability, may benefit from higher PEEP, whereas hypoinflammatory patients may be harmed by aggressive strategies.^[^^15^^]^ By incorporating these biological profiles, AI classifiers can move beyond generic optimization and provide strategies tailored to subgroups with different trajectories.

Personalization also requires combining static and dynamic inputs. Static inputs such as ARDS phenotype, predicted body weight, or comorbidities set the baseline context. Dynamic variables, including compliance trends, partial pressure of oxygen (PaO_2_) /fraction of inspired oxygen (FiO_2_) ratio, and asynchrony, dominate real-time decision-making. AI models integrate these layers so that long-term characteristics inform the safe range, while dynamic physiology drives breath-by-breath adjustments.^[^^6^^,^^9^^]^ This layered approach enables adaptive strategies individualized over time.

Beyond weaning and PEEP titration, personalization can extend to injury prevention. Continuous monitoring of driving pressure, mechanical power, or asynchrony index may help anticipate ventilator-induced lung injury and support early interventions.^[^^3^^,^^13^^]^ Recurrent models have already demonstrated the ability to predict respiratory failure hours in advance, enabling proactive interventions.^[^^8^^]^

## Conclusions

AI in mechanical ventilation is evolving from automated detection and protocolized support toward precision medicine. It can reduce workload, integrate multimodal signals, and improve patient–ventilator interaction. Translation into practice requires systems that are transparent, fault-tolerant, and capable of combining static features such as comorbidities or phenotypes with dynamic physiology such as compliance or oxygenation. The path forward involves embedding physiological rules, integrating ARDS subphenotypes, and incorporating fallback protocols to ensure safe recommendations.

Future implementation will depend on prospective validation within ARDS populations, where the integration of physiological principles and biological subphenotypes offers the clearest pathway toward personalized ventilation. Multicenter trials and collaborative databases will be essential to validate AI across populations, reduce bias, and assess clinical impact. Ultimately, we anticipate that AI-integrated systems will help reduce reintubation and ventilation duration in ARDS cohorts as predictive models continue to mature and integrate into bedside workflows. These developments are expected to facilitate the incorporation of AI-assisted strategies into future evidence-based guidelines. By combining physiological insights, biological profiles, and advanced learning methods, AI may finally deliver personalized ventilation strategies that anticipate lung injury or recovery while remaining interpretable and safe.

## CRediT authorship contribution statement

**Javier Muñoz:** Writing – original draft, Validation, Supervision, Project administration, Methodology, Investigation, Conceptualization. **Nerio José Fernández-Araujo:** Writing – review & editing, Formal analysis. **Rocío Ruíz-Cacho:** Writing – review & editing, Formal analysis. **Javier Muñoz-Visedo:** Writing – review & editing, Validation, Supervision, Formal analysis.
